# Exploring the structure of the university-students obsessive–compulsive tendency scale in Iranian university students: a network analysis study

**DOI:** 10.1186/s13104-023-06474-0

**Published:** 2023-09-04

**Authors:** Mohammadreza Davoudi, Mitra Sadoughi, Abbas Pourshahbaz, Behrooz Dolatshahi, Ali Nazeri Astaneh

**Affiliations:** 1 Department of Clinical Psychology, University of Social Welfare and Rehabilitation Sciences, Tehran, Iran; 2 Department of Educational Sciences, Tonekabon Branch, Islamic Azad University, Tonekabon, Iran; 3 Department of Psychiatry, University of Social Welfare and Rehabilitation Sciences, Tehran, Iran

**Keywords:** Obsessive–compulsive disorder, Obsessive–compulsive tendencies, Network Analysis

## Abstract

**Introduction:**

A risk factor for developing obsessive–compulsive disorder (OCD) in non-clinical samples is obsessive–compulsive tendencies (OCT). An OCT scale has recently been developed for university students (UOC) and showed promising psychometric properties. However, no validated Persian language scale evaluates OCT in non-clinical samples. Accordingly, this study aimed to validate the Persian version of the UOC in Iranian university students.

**Methods:**

Three hundred sixty-eight university students (54.6% females, mean ages: 22.4 ± 4.51) entered the study. The Persian version of UOC was evaluated concerning the structure of Exploratory Factor Analysis (EFA), Confirmatory factor analysis (CFA), and Exploratory graph analysis (EGA). Regarding the construct validity, the concurrent validity was assessed between the UOC and The Obsessive–Compulsive Inventory-Revised (OCI-R), Kessler Psychological Distress Scale (K10), and Yale-Brown Obsessive–Compulsive Scale (Y-BOCS). We calculated Cronbach’s alpha to evaluate the reliability of the UOC. All statistical calculations were done in R programming language (in R-Studio Desktop version 4.2.1).

**Results:**

The Persian version of UOC showed a convenient internal consistency with Cronbach’s alpha coefficient for the total scale 0.88. UOC scores were significantly correlated with OCI-R, K-10, and YBOCS. The EFA and EGA showed four and three-factor solutions with 25 and 28 items, respectively. Also, CFA showed that these two solutions were reliable, and the three-factors solution showed higher fit indexes. Finally, the results showed that item-27 was the most central item in the UOC network structure.

**Conclusion:**

The findings from the present study indicated that the Persian version of UOC has acceptable psychometric properties. So, this scale can be used for examining obsessive–compulsive tendencies in Iranian university students.

**Supplementary Information:**

The online version contains supplementary material available at 10.1186/s13104-023-06474-0.

## Introduction

Obsessive–Compulsive Disorder (OCD) is the most prevalent mental condition globally [1]. The lifetime prevalence of this psychiatric disorder in the general population was estimated between two and three percent. Recent research has shown that obsessive–compulsive symptoms are not exclusive to patients with OCD. Obsessive–compulsive symptoms have been investigated in the non-clinical population and named Obsessive–Compulsive Tendencies (OCT) [2]. Overall, previous studies support the idea that obsessive–compulsive symptoms are dimensional on a continuous spectrum. Accordingly, clinicians have reported that unwanted and intrusive thoughts are common in the non-clinical population [3]. The concept used to explain the behaviors and characteristics related to obsessive–compulsive symptoms in the general population (non-clinical) is OCT [4].

According to previous research, high levels of OCT correlate positively with hazardous cell phone usage, indecisiveness, and suicidal ideation [5, 6].

Regarding other psychiatric disorders, the results have shown that OCT can increase the severity of the disorder and the spread of psychological problems in psychiatric patients. For example, in a study, the results showed that patients with post-traumatic stress disorder who have higher levels of OCT demonstrated higher anxiety sensitivity, more severe PTSD symptoms, and higher levels of cognitive inflexibility than patients with PTSD without comorbid OCT [7]. Recently, a study found that more than 70% of university students show significant levels of OCT [8]. These findings are consistent with the continuum nature of obsessive–compulsive symptoms mentioned above. Therefore, it is necessary to investigate OCT in different populations, such as university students.

Recently, a scale was designed by Sashikata and Ozawa (2022) that examines Obsessive–Compulsive Tendencies in University Students (UOC) [9]. UOC was developed in an attempt to overcome the limitations of previous scales. Previous scales had extensive limitations. First, in the previous scales, hoarding was measured as one of the dimensions of OCD. First, in recent years, extensive studies have shown differences in regional brain activation, psychiatric comorbidities, and treatment outcomes between hoarding and OCD [10].

Additionally, The Diagnostic and Statistical Manual of Mental Disorders, Fifth Edition (DSM-5) now recognizes hoarding as a distinct syndrome called hoarding disorder. Second, most OCD-related scales (such as the Revised Obsessive–Compulsive Inventory and the Maudsley Obsessive–Compulsive Inventory) were based on clinical groups and may not be appropriate for measuring OCT in the general population [9, 11]. Finally, previous scales have not assessed indecisiveness, while results showed a strong correlation between indecisiveness and OCT [12].

Besides promising results about the development of UOC mentioned above, this scale development is facing a significant limitation. The authors investigated the psychometric properties of their scale based on the traditional approach. However, psychological variables are typically measured by assuming that the characteristics are latent variables (constructs) that are unobservable and influence observable behaviors. Without behavior-specific factors, these latent variables account for most of their variance and covariance. As a result, item scores reflect a person's position on a construct (hence the term 'reflective measurement models’). Extroverted or intelligent individuals will likely perform well on extraversion or intelligence tests. As a result, it begs the question of whether psychological constructs have a quantitative structure, if they exist independently of the test employed to evaluate them, and whether their variations affect the measurement process results.

In the past decade, latent factor models have been widely criticized in psychopathology. In latent factor models, symptoms are viewed as passive receptors for some common underlying factor independent of their local environment [13]. The DSM, which often reports causal relationships between symptoms related to the same disorder, does not even validate this assumption, which cannot be proven valid in most psychiatric disorders [14].

The network approach is an attractive alternative to the current common cause psychopathology model. Mental disorders can be conceptualized as a network system consisting of interconnected “elements” (nodes) in which changes in one node may lead to changes in the remaining nodes or, ultimately, changes in the entire network (*For more information, see *[13]).

It is only recently that these postulates have been applied to psychometrics. Several metrics have been developed for this situation, including network loadings, structural consistency, and exploratory graphs using face detection algorithms [15]. Further, a new generalization of network models has recently been proposed, allowing the assumption that latent networks are not intended to estimate causal relationships but interactions between variables [16]. The relationship between symptoms is an important aspect of the disorder, not the symptom itself. Each symptom (a node in the network) has different importance. There are more or less connections between a central node (or core node) [15]. Since central nodes impact other nodes directly, they play a more significant role in mental disorders. Due to the high risk of developing more severe OCD associated with core symptoms, items measuring core symptoms are more appropriate for OCT screenings.

Based on this, the present study aims to investigate this scale's psychometric characteristics by combining modern and traditional methods (network analysis) in Iranian university students (who speak the Persian language). The results of studies in Iran have shown that 34 and 54% of Iranian students have OCD [17, 18]. Meanwhile, the prevalence of OCD in students from other countries is much lower. For example, the prevalence of OCD in Indian and Saudi Arabia students was 3.3%, and 5.06%, respectively [19, 20].

Despite the high prevalence of obsessive–compulsive symptoms in Iranian students, there is no scale to evaluate OCT. The existence of such a scale can screen students with obsessive–compulsive symptoms at different stages of their studies. It is also possible to design interventions based on OCT in students to be used in the services of university counseling centers. This research aims to investigate the factorial structure of the Persian UOC applied to Iranian university students using established and novel (network psychometric) methodologies. The Persian version of this scale can help policymakers determine the dimensions of obsessive–compulsive tendencies in university students. Also, by accurately identifying the dimensions and severity of these tendencies in Iranian university students, interventions can be implemented to reduce the interference caused by them in universities. These measures are expected to increase the performance of Iranian students generally.

## Methods

### Design and participants

This cross-sectional study examines a scale’s psychometric properties and network structure among university students. We used convenience sampling (i.e., online advertisements) to collect data from all universities in Tehran, Iran. University students who voluntarily participated in our study were asked to complete a battery of web-based questionnaires. Sample size in validation studies recommends using a respondent-to-item ratio of at least 10:1 [21]. Since the UOC has 28 items, the sample size was estimated to be 280. However, about 30% was added to the sample size to reduce the potential bias. Therefore, the sample size increased to 368 subjects.

### Translation and cross-cultural adaptation

Necessary permissions for the translation adaptation study were obtained from the corresponding author who developed the questionnaire. The UOC translation and cross-cultural adaptation were conducted in five stages.

- Forward translation

Two native Persian speakers who were proficient in English translated the questionnaire from English into Persian. One of the translators who understood OCD was a Ph.D. candidate. An English instructor with 10 years of experience was the second translator, with neither a psychology background nor an awareness of OCD. An “A” and “B” translation version was produced.

- Translation synthesis

The authors met with the two translators to synthesize “A” and “B.” As a result, a single questionnaire (C) was created.

- Backward translation

A Persian version of the UOC (C) was synthesized by two other English translators proficient in Persian and then translated back into English by two other English translators. The translators did not have a behavioral sciences background. Also, they had no access to UOC or similar scales. Additionally, they were asked not to search for the questionnaire. “X” and “Y” were two back translation versions.

- **Expert committee**

In addition to being a methodologist with a Ph.D. in psychometrics and a specialization in epidemiology and biostatistics, two authors and translators also served on the expert committee. They compared the back-translated and original versions of the UOC to identify discrepancies or inconsistencies in the translation process. The UOC's pre-final Persian version was revised to ensure clarity and suitability for a general Persian audience.

- **Pilot testing**

Thirty psychology students were asked to identify ambiguous items in their responses using the tool. Clinical psychologists and psychiatrists with doctoral degrees developed, revised and approved the tool. As a result of the amendments received, the Persian version included questions ranging from not very much [1] to very much [6].

### Measures

#### Descriptive variables

A researcher-made scale includes age, gender, education level, marital status, medical history, and psychiatric history, used to report their descriptive statistics.

#### The obsessive–compulsive inventory-revised (OCI-R)

A self-report scale includes 18 items to evaluate obsessive–compulsive symptoms. Participants reported their past-month obsessive–compulsive distress on a five-point Likert scale (zero = not by any means to four severely). This scale includes Washing Concerns, Mental Neutralizing, Checking/Doubting, Obsessing, Hoarding, and Ordering. If in each subscale, the score of the participants is above “seven,” the person has a significant (dominant) subtype. The Persian version of this scale showed suitable test–retest reliability (from 0.62 to 0.76) and internal consistency (from 0.77 to 0.86) for OCI-R subscales. Finally, the subscale correlations were estimated from 0.51 to 0.76 [22].

#### The Yale-brown obsessive–compulsive scale (YBOCS)

A rated scale, clinician-administered, and semi-structured interview to evaluate symptom severity and to recognize the type of obsessions present. The YBOCS has five items for obsessions and five items for compulsions. According to Esfahani et al., the internal consistency, split-half reliability, and test–retest reliability were calculated at 0.97, 0.93, and 0.99, respectively. Also, they found that this scale significantly correlates with Symptom Checklist-9 [23]. We used a self-report version of YBOCS in the Persian language.

#### Obsessive–compulsive tendencies scale of students

This tool is designed to examine OCT in students. This tool examines obsessive tendencies in 5 dimensions on a 5-point Likert scale (1 = never to 5 = extremely). Obsession, Indecision, Ordering, Cleaning, and Checking are the five subscales. The results of the original version of this tool showed that this tool has good psychometric characteristics. The psychometric properties of the Persian version of this scale are presented in the result section.

#### Kessler psychological distress scale (K10)

The K10 is a self-report scale to evaluate psychological distress. The K10 scale consists of 10 emotional state-related questions, each with a five-level answer scale. The test can be used as a quick screening tool to determine the severity of discomfort. Patients can complete the form independently or with the practitioner's help, who can also read the questions. In the previous studies, the Cronbach alpha and split-half reliability of the Persian version of K10 was estimated at 0.92 and 0.85, respectively [24].

### Procedures

A battery of mentioned scales was created for data collection between September to November 2022. The questionnaire was shared through the Porsline form (an Iranian website for surveys), and researchers shared the online link. This link was posted along with an advertising poster of participating in the research in the Telegram channels (the most used social media in Iran) of universities in Tehran (with more than a thousand students). In this poster, students were asked to answer these questions if they were studying at one of the universities in Tehran. To increase the response rate, the researchers explained to the students the prevalence of obsessive tendencies in university students and its effect on their declining quality of life. The researchers expected participants to respond to each survey item within two seconds [25]. The online survey consisted of consent, an introduction to each questionnaire, 73 items, and a short confirmation. We excluded participants who provided invalid responses (i.e., the same answer to all items) or completed the battery in less than four minutes [26]. We surveyed 368 university students from a sample of 527. A total of 69.8% of respondents completed the questionnaire. Incomplete questionnaires were excluded from the analysis**.**

### Statical analysis

R-software (version 4.2.1) carried out statistical analysis in seven basic steps.

#### Descriptive statistic

In the first step, basic packages of R (base R) were used to describe the data. In this section, demographic variables (e.g., age, gender, education level) and clinical variables (mean and standard deviation of scales) were described.

#### Network estimation

Initially, we used the “qgraph” R package for network estimation. The “qgraph” R package allows for identifying patterns by uniquely displaying data: through network visualization (For more info ([27])). Before conducting the analysis, the data underwent a standardization transformation process. Additionally, we estimated centrality measures for identifying significant nodes. Centrality is utilized to gauge the significance of different nodes. Each node may be considered significant from different perspectives, depending on how “importance” is defined. Different centrality measures are sensitive to various aspects of the relationships between a focal unit and other units. Our study calculated three standard centrality measures used in behavioral sciences: Betweenness, Closeness, and Strength (For more information, see [28]). The estimates are shown in a Z-standardized format. Greater values suggest greater centrality.

Furthermore, we calculated a new measure of centrality known as “expected influence.” The expected influence (EI) is characterized by the total number of a node's linkages and presents its significance in the network. This importance is relative because, even in networks with low overall edge weights, there will always be a node with strong expected influence, assuming standardized outcomes [27].

#### Exploratory factor analysis (EFA)

We used psych, corrplot, psych, ggplot2, car, and huge packages to evaluate EFA [29–31]. After cleaning the data, we estimated the correlation matrix for UOC items. We also should look at the correlations among our variables to determine if factor analysis is appropriate. Then Factorability is better assessed using the Kaiser–Meyer–Olkin (KMO) method, which is also used to assess sampling adequacy. Kaiser recommends using KMO ≥ 60 to assess the factorability of the sample data [32]. Finally, the factor analysis and screen plot were performed using the packages above.

#### Exploratory graph analysis (EGA)

As a method for estimating weighted networks, EGA uses a network estimation algorithm with a community detection algorithm [15]. The network is estimated using a Gaussian graphical model using the qgraph package, which implements an extended Bayesian information criterion model selection method in conjunction with the Least Absolute Shrinkage and Selection Operator (LASSO) regularization procedure. LASSO uses the correlation matrix of the observable variables to generate a sparse inverse covariance matrix. Using the Walktrap algorithm with the igraph package, the number of dense subgraphs is specified after computing the partial correlation matrix. Walktrap involves “random walks” between nodes. The probability of walking between two nodes increases when they are closely correlated. As a result, node modules are highly connected.

#### Confirmatory factor analysis (CFA)

The multifactor solution obtained using the traditional method (EFA) and the one obtained using the network method (EGA) were compared. Amos analyzed both cases statistically. The model fit was evaluated using maximum likelihood estimation, along with the χ2, comparative fit index (CFI), Tucker-Lewis index (TLI), and root-mean-square error of approximation (RMSEA). Acceptable values were CFI/TLI ≥ 0.90, RMSEA ≤ 0.08, and p < 0.05 for χ2. Using the Akaike information criterion (AIC), we assessed the fit and parsimony of the two models. The smaller the value, the better the fit [33].

#### Reliability

This statistic can be used to determine if a collection of items consistently measures the same characteristic. To determine Cronbach's Alpha agreement level, “***itm***,” the R-studio package for measuring Cronbach's Alpha uses a standardized 0–1 scale. Cronbach's Alpha agreement level can be evaluated using a standardized 0–1 scale.

#### Convergent and divergent validity

Convergent validity tests confirm relationships between notions that are predicted to be connected. Divergent validity (also known as discriminant validity) examines whether constructs that ought to be unrelated are, in fact, unrelated. For divergent and convergent validity estimates in R-studio, we employed Pearson correlation.

## Results

### Descriptive statistics

Table 1 displays the participant’s demographic information. Three hundred sixty-eight university students aged 17 to 47, with an average age of 22.4 years, participated in this study. (SD: ± 4.51). Also, Additional file 2: Table.S1 presented the descriptive statistics of the Persian version of UOC items (Mean, Standard Deviation, Skewness, and Kurtosis).

**Table 1:** Demographic and clinical characteristics of the participants

Characteristics	N	N(%)/mean (± SD)
Age		22.4 ± 4.51
Gender	Male	167(45.38%)
	Female	201(54.62%)
Marital status	Single	305(82.8%)
	Married	15(4.1%)
	In a romantic relationship	44(12%)
	Widow/Divorced	4(1.1%)
Educational status	Associate of science	5(1.4%)
	Bachelor's degree	254(69%)
	Master’s degree	48(13%)
	Medical doctor/ PhD	61(16.6%)
Economical level	Very low	46(12.5%)
	Low	63(17.11%)
	Medium	117(31.8%)
	High	98(26.64%)
	Very high	44(11.95%)
OCI-R		26.74(± 10.58)
K10		25.36(± 8.38)
Y-BOCS		10.2(± 4.3)
UOC		64.04(± 19.30)

### The network estimation

**Fig. 1:**
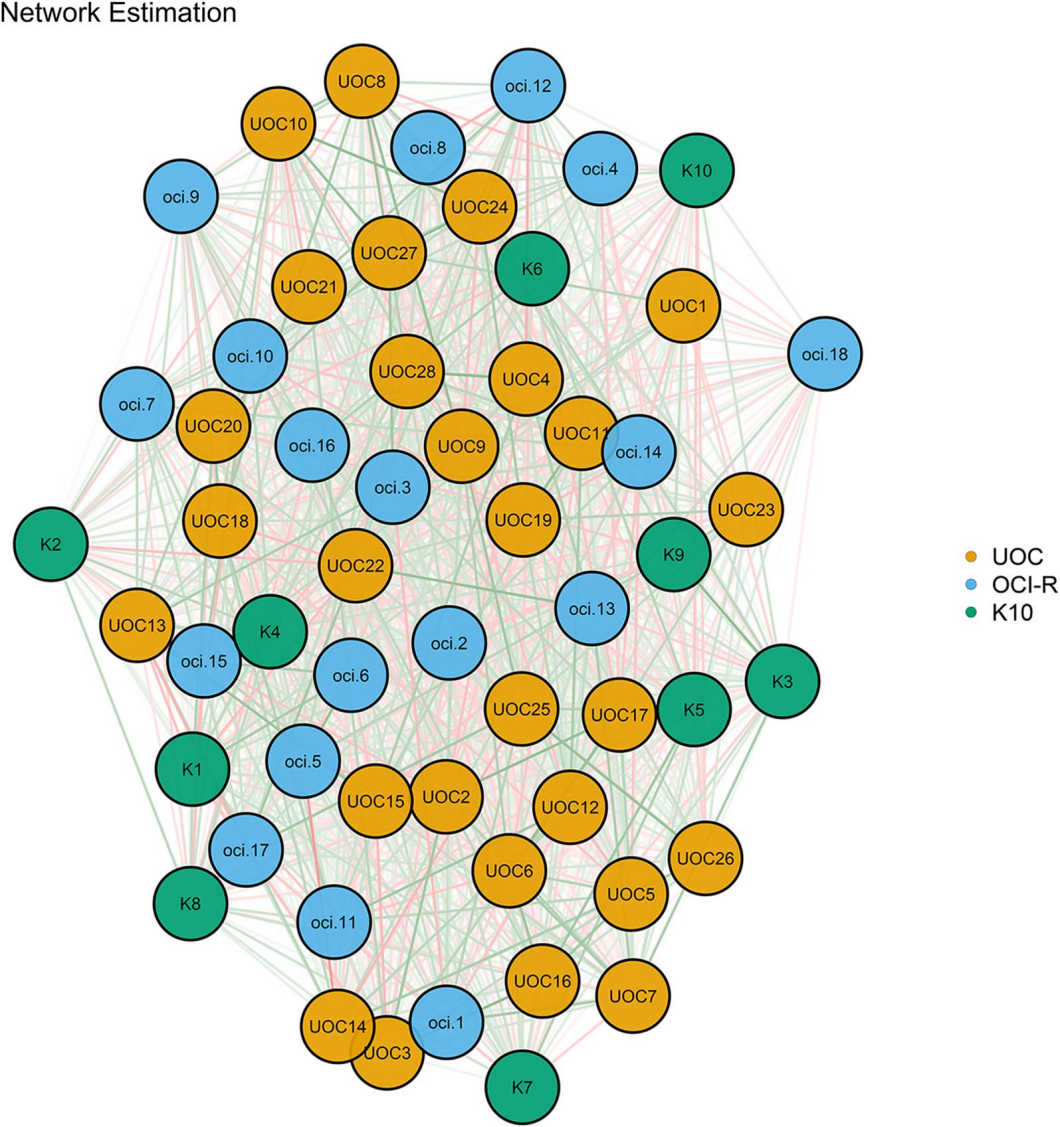
The network structure in participants. Reds = Negative Correlations, Green = Positive Associations. Thicker lines = Stronger relationships

First, the network of symptoms was estimated for all clinical scales. Figure 1 shows the network structure of variables in all participants (short names were defined in Additional file 2: Table S2).

**Fig. 2:**
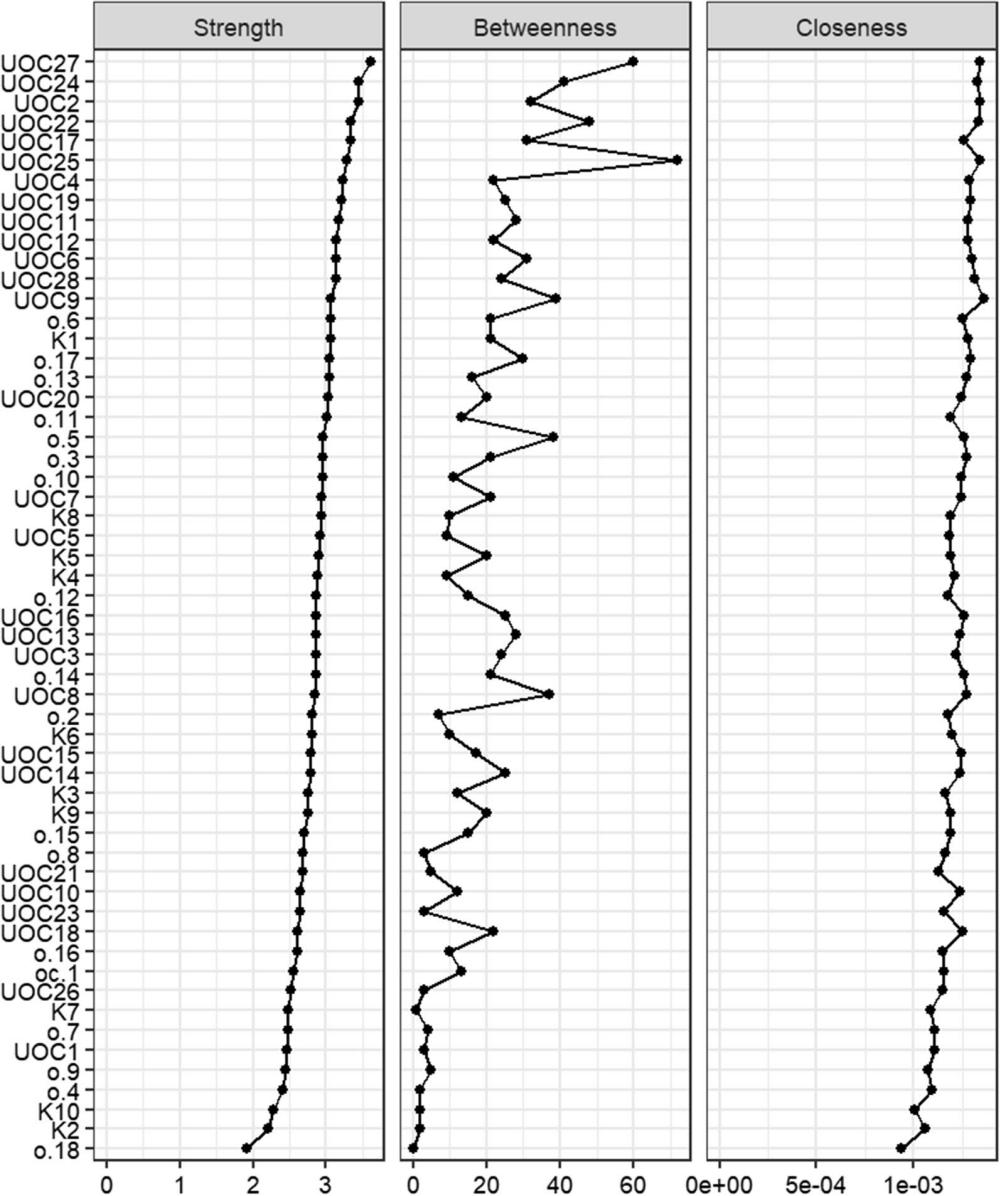
Centrality measures for Strength, Closeness, Betweenness, and ExpectedInfluence

Figure 2 presents the visual perspective of centrality measures separately for each scale. This work will reduce unnecessary complexity and provide a more accurate interpretation of the results. Table.S3 gives the exact numerical value of these centrality measures separately for the scales. Regarding the centrality measures, UOC-27 (I feel uneasy when the things I see are not clean) showed high scores in all three centrality measures (strength = 2.313272805, Betweenness = 2.766499539, and Closeness = 1.39978134). Also, centrality measures showed in the Additional file (Additional file 2: Table S3). About EI results showed that the UOC25, UOC27, and UOC16 had the highest scores, in order (see Additional file 2: table S3 for exact numbers).

### Exploratory factor analysis (EFA)

Our data were first analyzed to ensure that conducting EFA would be appropriate. We evaluated the KMO model for this purpose (Overall = 0.9). Bartlett's sphericity test resulted in a value of 0.899 and P < 0.01, proving that our matrix was suitable for the calculations. Following numerous factor solutions, the four-factor solution showed the best fitness (Table 2). According to the EFA findings, only the four-factor solution produces eigenvalues more significant than one, particularly 3.88. The four-factor model explains 43.15% of the total variance. Positive factorial loads greater than 0.42 were present for all items. The study revealed four dimensions. Therefore, the answer was rotated using a varimax. The EFA output also removed items 1, 13, and 24. These four components were identified as the factors in this questionnaire using the exploratory factor analysis. The first subscale is cleaning, the second is indecisiveness, the third has obsessions, and the fourth is checking, according to the values. Also, Fig. 1S presents the eigenvalues of components in the screen plot. Finally, we estimated the correlation matrix for UOC items, and the results showed that this scale has a suitable correlation (see Fig. 2S).

**Table 2:** Exploratory factor analysis for the Persian version of the scale

Items	cleaning^1^	indecisiveness ^2^	Obsessions^3^	Check^4^
8	0.712			
10	0.694			
18	0.640			
20	0.617			
25	0.521			
27	0.722			
9	0.422			
22	0.489			
2		0.604		
3		0.524		
5		0.607		
6		0.653		
7		0.589		
16		0.625		
17		0.656		
14			0.595	
15			0.530	
19			0.529	
23			0.609	
26			0.528	
21			0.487	
4				0.614
11				0.665
12				0.712
28				0.668

### Exploratory graph analysis (EGA)

**Fig.3:**
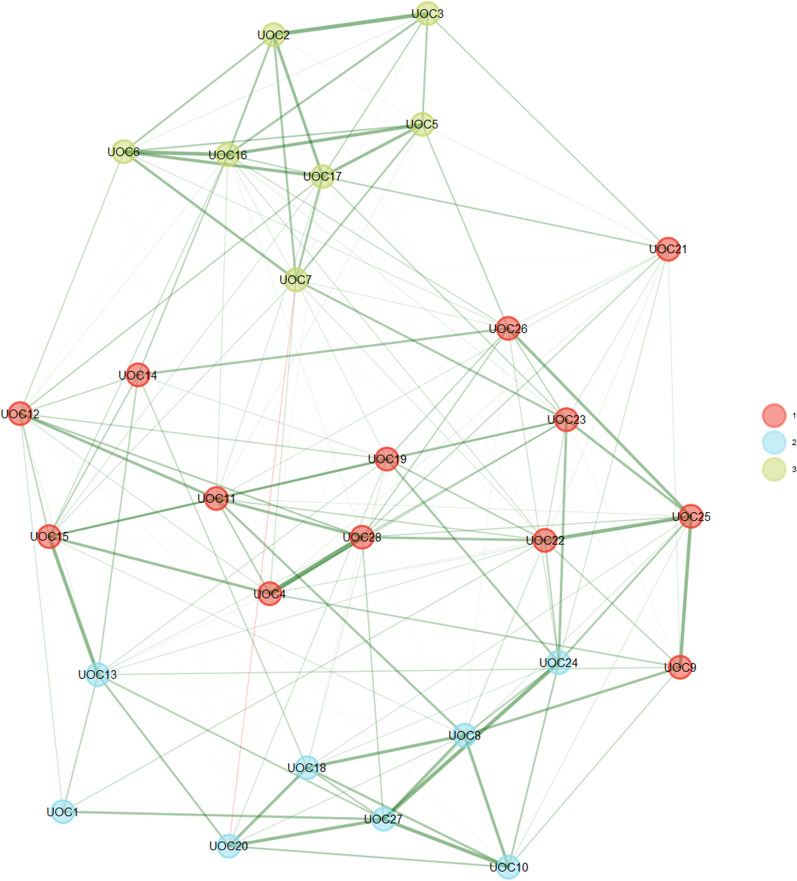
Dimensionality Results for EGA for the UOC Scale. The LASSO algorithm, which assessed the model based on partial relationships and used the penalty approach to produce sparser networks, was used to estimate the EGA. The median network structure reflects the same dimensions determined by EGA

EGA estimated the three-dimensional structure of the scale (Figure 3, Table 3). The first dimension comprises Items 4,9,21,26, 14,12, 15,11,19,23,25,22, and 28. The second dimension includes items 1,8,10,13,18,20,24, and 27. Finally, the third dimension includes items 2,3,5,6,7,16 and 17. After item analysis, the first dimension is named “general obsessions,” and the second factor is named contamination/cleaning. Finally, results showed that the third factor evaluated “Indecisiveness.” The median network structure (Figure.3 and Table.3) reflects the same dimensions estimated via EGA, with a relatively narrow confidence interval CI [95% CI (1.87, 4.152)].

**Table 3:** Descriptive statistics of the Persian version of UOC dimensions across all bootstrap replicate samples

n.Boots	Median.dim	SE.dim	CI.dim	Lower.CI	Upper.CI	Lower quantile	Upper. quantile
1000	3	0.5874	1.152	1.847	4.152	3	5

According to the frequencies, 799 out of 1,000 bootstrapped samples yielded three dimensions in 79.9% of the cases (Table 4). Numerous other dimensional structures also appeared, particularly one with four dimensions (around 14.2% of the time), which suggests that the four-dimensional solution is probably unstable. Therefore, three dimensions appeared to be the most stable solution.

**Table 4:** Frequency of the Persian version of UOC dimensions across all bootstrap replicate Samples

# Of dimension	Frequency
Two	0.001
Three	0.799
Four	0.142
Five	0.050

### CFA

As shown in Table 5, CFA was used to analyze the results of EGA (all UOC items and three-factors solution) and EFA (including 25 items and four-factors solution); for EFA, items 1,13, and 24 were excluded, but all items, were included in the EGA analysis. According to Table 3, both models are relatively similar. However, the EGA solution generally showed greater values in model fit indexes.

**Table 5:** Model fit statistics for the UOC

	Method	χ2	df	p Value	CFI	TLI	RMSEA	CMIN/DF	AIC
1	(Solution EFA)	491.468	269	< 0.001	0.903	0.892	0.086	1.82	653.46
2	(Solution EGA)	526.098	347	< 0.001	0.916	0.909	0.041	1.62	763.09

### Reliability

Cronbach's alpha was used to estimate the UOC's reliability. Internal consistency, or how closely connected a group of things are to one another, is measured by Cronbach's alpha. Cronbach's alpha was estimated at 0,88, according to EGA items. This amount for the EFA solution (three items were removed in this measure) included 0.87.

### Convergent and divergent validity

The relationships between the Persian version of the UOC and other scales used to determine the divergent and convergent validity of the concept are shown in Table 6. All other scales have a positive relationship with the UOC. Also, we used an EGA factor solution to deter the association between UOC subscales with OCD severity, psychological distress, and OCD dimension.

**Table 6:** Convergent and divergent Validity for U-OCT (total score and their subscales)

U-OCT	OCI. Hoarding	OCI. ordering	OCI. washing	OCI. checking	OCI. neutralization	OCI. obsession	YB.Total	K. total
Total scores	0.553^**^	0.350^**^	0.570^**^	0.520^**^	0.534^**^	0.308^**^	0.662^**^	0.484^**^
General obsessions	0.54^**^	0.375^**^	0.568^**^	0.567^**^	0.573^**^	0.311^**^	0.636^**^	0.374^**^
Contamination/cleaning	0.360^**^	0.366^**^	0.427^**^	0.349^**^	0.367^**^	0.086	0.359^**^	0.205^**^
Indecisiveness	0.354^**^	0.112^*^	0.291^**^	0.235^**^	0.282^**^	0.276^**^	0.296^**^	0.294^**^

## Discussion

This study investigated the psychometric properties of Persian OC tendencies among Iranian university students. To assess the factorial structure of the UOC, a methodology derived from the network approach was used in addition to traditional statistical analyses, such as EFA and EGA. To provide evidence for the factorial structure of the questionnaire, the study sought to gain a deeper understanding of self-reporting. To this end, a sample of Iranian university students studying at one of the universities in Tehran, Iran, was used. The original version was translated and back-translated for appropriate adjustment. The expert committee modified the translated version, and finally, the latest version was executed in pilot form and modified. EFA, EGA, CFA, reliability (internal consistency), and validity (convergent and divergent) were explored in sequence for this scale. First, the EFA results indicated that the data fit best with the four-factor structure of the Persian version of the UOC tendencies scale. Items 1, 13, and 24 of this scale were omitted because of poor factor loading. These factors include cleaning, indecisiveness, obsessions, and checks. However, the original version found five factors similar to our results except for "ordering."

Second, the EGA results indicated that the data fit best with the three-factor structure of this scale. Also, in this solution, all items of the original version were included. CFA estimates show that the three-factors solution (EGA estimation) had better indexes than the four-factors solution (EFA estimation). Three factors in EGA estimation were “general obsessions,” “contamination/cleaning,” and “Indecisiveness.” Third, internal consistency was estimated by Cronbach's alpha.

The results showed that the 28-item Persian version of the UOC tendencies scale had high internal consistency (α = 0.88). Finally, our results showed that higher UOC tendency scores were positively correlated with higher levels of obsessive–compulsive severity, psychological distress, and obsessive–compulsive dimensions. In the original version (Japanese university students), this scale included five subscales, while the present study (Iranian university students) identified three subscales [9]. In fact, in the original version, there are two scales, including Ordering and Obsessions, which were not identified in the Persian version of this scale. This discrepancy can have several reasons. First, in the original scale, the items related to ordering and cleaning subscales had relatively similar content. For example, one of the items related to order was "*I have to keep my things and room clean*." As it is clear from the content of this scale, this scale includes content between ordering and cleaning. Therefore, the content overlap between the scales can be one of the reasons for this disparity in findings between the original research and the current study. Second, recent studies have shown that culture can be essential in the phenomenology of obsessive–compulsive symptoms. Culture can affect the severity, prevalence, and manifestations of obsessive–compulsive symptoms [34].

Third, when the traditional method of measurement (EFA) was used, four subscales were obtained, including cleaning, indecisiveness, Obsessions, and Check. Therefore, the EFA results were more similar to the original study’s. In another research conducted by Li and Shimoyama [11], results showed a four-factor solution for OCT, including checking, ordering, doubt/control, and cleaning among Chinese university students. In total, these three studies show a difference in the factor structure of this tool in different countries (at the same time, the optimal psychometric properties of this scale are different in all three countries). Therefore, this difference in the factor structure can reflect some cultural differences in obsessive–compulsive tendencies. Based on this, repeating this study in other countries can evaluate the role of culture in the phenomenology of these symptoms.

The divergent/convergent validity results highlighted that higher OCT was significantly associated with higher levels of OCD severity, psychological distress, and OCD dimensions. This finding shows that there may be an association between OCT and various psychological issues that should be researched to enhance university students’ mental health. These results were in line with previous studies. For example, Sashikata and Ozawa found that OCT was significantly correlated with obsessive–compulsive symptoms (which were assessed by OCI-R) and psychological distress [9]. Also, Lester et al. evaluated OCT in undergraduates. They found that OCT was significantly associated with depression and suicidal tendencies [35]. These findings align with our results that OCT correlated with psychological distress.

On the other hand, the results showed that Indecisiveness as part of the content of OCT items significantly correlates with the symptoms and severity of OCD. These results have been replicated in different populations, including children, youth, and adults [36, 37]. Our results showed that Indecisiveness is associated with hoarding (one OCI-R dimension). This result was in line with previous studies. For example, Frost et al. found that individuals with hoarding problems had more decision-making problems (Indecisiveness) [38]. Finally, according to our results, Cronbach’s alpha (internal consistency) was estimated at 0.88. This value is highly similar to Cronbach alpha values in the original version of UOC (0.81–0.87 for subscales). According to various estimations, Cronbach values of 0.80 or above are preferred [39]. Finally, the Persian version of this scale is accessible upon reasonable requests.

## Conclusion

Our research aimed to estimate the factor structure of the Persian version of OUC in Iranian university students. Our findings support the three-factor approach, which is also supported by network methodology (EGA) and conventional statistics (CFA), even though both models produce reasonable results. On the other hand, considering the high prevalence of OCD and its symptoms in Iranian students, this tool can be used as a scale for screening obsessive–compulsive symptoms/tendencies in Iranian university students. Also, policymakers in the Ministry of Health can identify the risk factors related to OCT and design related protocols to be implemented in counseling centers of universities for vulnerable students.

## Limitations

Besides promising results, some limitations need to be addressed. First, face and content validity were not investigated in the present study. Second, the studies in which EGA has been used in examining the psychometric characteristics of scales related to behavioral sciences are relatively few. Third, in this study, the authors used a cross‐sectional design, and the results could not estimate the directions of effects. Finally, this study was conducted during the COVID-19 pandemic time. So, we were forced to data gathering with an online platform. This can potentially reduce the generalizability of the present results. So, conducting future studies with in-person data collection can provide a better perspective. Also, future studies with other network analysis tools should be used to assess symptoms’ predictability. Also, the role of UOC in academic performance and student quality of life could be evaluated in future studies.

### Supplementary Information

**Additional file 1:** Screen plot for UOC and Correlation Matrix For UOC Items.

**Additional file 2: Table S1.** Descriptive statistic of UOC scale. **Table S2.** Full items names with short names definitions. **Table S3.** Centrality Measures of Network structure (Network analysis of all variables).

## Data Availability

The corresponding author will provide the data sets obtained and/or evaluated within the current work upon reasonable request.

## Abbreviations

EFA: Exploratory factor analysis
OCD: Obsessive–compulsive disorder
LASSO: Least Absolute Shrinkage and Selection Operator
K10: Kessler psychological distress scale
CFI: Comparative fit index
RMSEA: Root‐mean‐square error of approximation
UOC: Obsessive–compulsive tendencies in university students scale
OCI-R: The Obsessive–Compulsive Inventory-Revised
OCT: Obsessive–compulsive tendencies
EGA: Exploratory graph analysis
YBOCS: The Yale-Brown Obsessive–Compulsive Scale
TLI: Tucker–Lewis index
DSM-5: The Diagnostic and Statistical Manual of Mental Disorders, Fifth Edition
