# Supplementary material for: Exploring the structure of the university-students obsessive–compulsive tendency scale in Iranian university students: a network analysis study
Source: BMC Res Notes. 2023 Sep 4;16:193. doi: 10.1186/s13104-023-06474-0 (PMC10476335; doi:10.1186/s13104-023-06474-0)
Supplement: Supplementary file 2 — Additional file 2: Table S1. Descriptive statistic of UOC scale. Table S2. Full items names with short names definitions. Table S3. Centrality Measures of Network structure (Network analysis of all variables). [file 13104_2023_6474_MOESM2_ESM.docx]

| Table S1. Descriptive statistic of UOC scale | | | | | | |
| --- | --- | --- | --- | --- | --- | --- |
|  | mean | sd | min | max | skew | kurtosis |
| UOC1 | 2.79 | 1.42 | 0 | 5 | -0.09 | -0.97 |
| UOC2 | 2.94 | 1.42 | 0 | 5 | -0.07 | -1.16 |
| UOC3 | 2.97 | 1.44 | 0 | 5 | 0 | -1.26 |
| UOC4 | 2.3 | 1.6 | 0 | 5 | 0.19 | -1.12 |
| UOC5 | 2.66 | 1.54 | 0 | 5 | 0.03 | -1.16 |
| UOC6 | 2.76 | 1.55 | 0 | 5 | 0.03 | -1.25 |
| UOC7 | 2.69 | 1.6 | 0 | 5 | -0.01 | -1.2 |
| UOC8 | 2.3 | 1.59 | 0 | 5 | 0.25 | -1.15 |
| UOC9 | 2.57 | 1.58 | 0 | 5 | 0.03 | -1.17 |
| UOC10 | 2.52 | 1.47 | 0 | 5 | 0.1 | -1.08 |
| UOC11 | 2.4 | 1.6 | 0 | 5 | 0.23 | -1.07 |
| UOC12 | 2.55 | 1.45 | 0 | 5 | 0.12 | -1.02 |
| UOC13 | 2.62 | 1.51 | 0 | 5 | 0.07 | -1.06 |
| UOC14 | 2.86 | 1.47 | 0 | 5 | -0.06 | -1.18 |
| UOC15 | 2.54 | 1.49 | 0 | 5 | 0.07 | -1.03 |
| UOC16 | 2.67 | 1.55 | 0 | 5 | 0.11 | -1.18 |
| UOC17 | 2.73 | 1.63 | 0 | 5 | -0.03 | -1.29 |
| UOC18 | 2.55 | 1.57 | 0 | 5 | 0.13 | -1.13 |
| UOC19 | 2.6 | 1.55 | 0 | 5 | 0.06 | -1.17 |
| UOC20 | 2.68 | 1.42 | 0 | 5 | 0 | -1.05 |
| UOC21 | 2.61 | 1.43 | 0 | 5 | 0.14 | -1 |
| UOC22 | 2.23 | 1.68 | 0 | 5 | 0.21 | -1.19 |
| UOC23 | 2.45 | 1.62 | 0 | 5 | 0.09 | -1.17 |
| UOC24 | 2.15 | 1.56 | 0 | 5 | 0.43 | -0.95 |
| UOC25 | 2.2 | 1.68 | 0 | 5 | 0.29 | -1.13 |
| UOC26 | 2.45 | 1.55 | 0 | 5 | 0.2 | -1.08 |
| UOC27 | 2.52 | 1.56 | 0 | 5 | 0.12 | -1.11 |
| UOC28 | 2.3 | 1.66 | 0 | 5 | 0.22 | -1.19 |

| **Table S2. Full items names with short names definitions.** | |
| --- | --- |
| Items Full name. | Abbreviations |
| I have to keep things on the table clean. | table clean |
| I sometimes become anxious when I have to make decisions. | anxious decisions |
| I sometimes worry about the decisions I have made. | worry decisions |
| I sometimes have to return home to check things (e.g. door or windows drawer, etc.) to make sure they are properly shut find. | return home to check |
| It difficult to make decisions when I see the menu and order or go shopping. | make decisions order |
| I am slow in making decisions. | Slow decisions |
| I sometimes cannot pay attention to what is happening around me because my mind starts thinking certain things automatically. | Pay |
| I think even slight contact with my body (perspiration saliva urine etc.) may contaminate clothes or somehow harm me | contact harm |
| I feel my hands are dirty after touching money. | dirty money |
| If I touch something I think is dirty I immediately have to wash or clean myself. | touch wash |
| I sometimes keep on checking things more often than necessary. | checking often |
| I sometimes have to do things several times before I think they are properly done. | several time" |
| I feel uneasy when books are not lined up neatly on the bookshelf. | uneasy bookshelf |
| I sometimes worry about losing my things or forgetting something. | worry forgetting |
| I sometimes check several times when I do something. | check something |
| I sometimes check several times when I do something. | check several |
| I hate being in a situation where I have to decide something. | hate decides |
| I feel dirty when I touch animals and I immediately want to wash my hands or change my clothes. | dirty animals |
| When I start thinking of certain things, I sometimes become obsessed with them. | thinking obsessed |
| I have to keep my things and room clean. | room clean |
| When unpleasant thoughts come into my mind, I sometimes cannot get rid of them. | unpleasant thoughts |
| I feel unclean when I accidentally bump into somebody in the train. | bump train |
| I sometimes worry about losing control and doing disturbing things. | worry control |
| I sometimes worry for no reason that I have some disease. | worry disease |
| I find it difficult to touch something when I know it has been touched by someone. | touch touched", |
| I have feelings of distrust and doubt about most of my actions. | distrust actions |
| I feel uneasy when the things I see are not clean. | I uneasy clean |
| I sometimes check and recheck gas water taps and doors. | check gas |

| Table 3S. Centrality Measures of Network structure (Network analysis of all variables) | | | | | | | | | |
| --- | --- | --- | --- | --- | --- | --- | --- | --- | --- |
|  | node | measure | value | measure | value | measure | value | measure | value |
| 1 | UOC1 | Betweenness | -1.1083 | Closeness | -1.25973 | Strength | -1.22465 | ExpectedInfluence | -1.55368 |
| 2 | UOC2 | Betweenness | 0.86309 | Closeness | 1.416921 | Strength | 1.78587 | ExpectedInfluence | -0.05329 |
| 3 | UOC3 | Betweenness | 0.319258 | Closeness | -0.00653 | Strength | -0.00071 | ExpectedInfluence | -0.83494 |
| 4 | UOC4 | Betweenness | 0.1833 | Closeness | 0.845649 | Strength | 1.134116 | ExpectedInfluence | 0.153913 |
| 5 | UOC5 | Betweenness | -0.70043 | Closeness | -0.34268 | Strength | 0.171372 | ExpectedInfluence | -0.08324 |
| 6 | UOC6 | Betweenness | 0.795111 | Closeness | 0.934415 | Strength | 0.83941 | ExpectedInfluence | -0.38663 |
| 7 | UOC7 | Betweenness | 0.115321 | Closeness | 0.358837 | Strength | 0.22904 | ExpectedInfluence | 1.49792 |
| 8 | UOC8 | Betweenness | 1.202984 | Closeness | 0.687125 | Strength | -0.08356 | ExpectedInfluence | 0.519017 |
| 9 | UOC9 | Betweenness | 1.338942 | Closeness | 1.684778 | Strength | 0.621272 | ExpectedInfluence | 0.09623 |
| 10 | UOC10 | Betweenness | -0.49649 | Closeness | 0.286799 | Strength | -0.65587 | ExpectedInfluence | 0.017992 |
| 11 | UOC11 | Betweenness | 0.591174 | Closeness | 0.751343 | Strength | 0.923755 | ExpectedInfluence | 0.790206 |
| 12 | UOC12 | Betweenness | 0.1833 | Closeness | 0.700163 | Strength | 0.860533 | ExpectedInfluence | 0.506535 |
| 13 | UOC13 | Betweenness | 0.591174 | Closeness | 0.246404 | Strength | 0.008364 | ExpectedInfluence | 1.048993 |
| 14 | UOC14 | Betweenness | 0.387237 | Closeness | 0.261857 | Strength | -0.25008 | ExpectedInfluence | -0.37267 |
| 15 | UOC15 | Betweenness | -0.15659 | Closeness | 0.358812 | Strength | -0.22116 | ExpectedInfluence | 0.090331 |
| 16 | UOC16 | Betweenness | 0.387237 | Closeness | 0.527045 | Strength | 0.013733 | ExpectedInfluence | 1.526429 |
| 17 | UOC17 | Betweenness | 0.795111 | Closeness | 0.525967 | Strength | 1.434588 | ExpectedInfluence | 1.259618 |
| 18 | UOC18 | Betweenness | 0.1833 | Closeness | 0.421034 | Strength | -0.76677 | ExpectedInfluence | -0.18897 |
| 19 | UOC19 | Betweenness | 0.387237 | Closeness | 0.852637 | Strength | 1.071337 | ExpectedInfluence | 1.28516 |
| 20 | UOC20 | Betweenness | 0.047342 | Closeness | 0.303056 | Strength | 0.520263 | ExpectedInfluence | -0.61848 |
| 21 | UOC21 | Betweenness | -0.97234 | Closeness | -0.97007 | Strength | -0.57205 | ExpectedInfluence | 0.752352 |
| 22 | UOC22 | Betweenness | 1.950752 | Closeness | 1.331683 | Strength | 1.451619 | ExpectedInfluence | 1.202832 |
| 23 | UOC23 | Betweenness | -1.1083 | Closeness | -0.6867 | Strength | -0.68811 | ExpectedInfluence | 0.650113 |
| 24 | UOC24 | Betweenness | 1.4749 | Closeness | 1.247173 | Strength | 1.808207 | ExpectedInfluence | 0.276645 |
| 25 | UOC25 | Betweenness | 3.582247 | Closeness | 1.414332 | Strength | 1.277241 | ExpectedInfluence | 2.439183 |
| 26 | UOC26 | Betweenness | -1.1083 | Closeness | -0.76671 | Strength | -1.0527 | ExpectedInfluence | 0.152912 |
| 27 | UOC27 | Betweenness | 2.7665 | Closeness | 1.399781 | Strength | 2.313273 | ExpectedInfluence | 2.325471 |
| 28 | UOC28 | Betweenness | 0.319258 | Closeness | 1.160267 | Strength | 0.829707 | ExpectedInfluence | 1.007434 |
| 29 | OCI-R1 | Betweenness | -0.42851 | Closeness | -0.6798 | Strength | -0.94237 | ExpectedInfluence | -0.47447 |
| 30 | OCI-R2 | Betweenness | -0.83638 | Closeness | -0.47238 | Strength | -0.16171 | ExpectedInfluence | -0.13562 |
| 31 | OCI-R3 | Betweenness | 0.115321 | Closeness | 0.627543 | Strength | 0.278705 | ExpectedInfluence | -1.77553 |
| 32 | OCI-R4 | Betweenness | -1.17628 | Closeness | -1.3493 | Strength | -1.43079 | ExpectedInfluence | 0.472229 |
| 33 | OCI-R5 | Betweenness | 1.270963 | Closeness | 0.470876 | Strength | 0.280294 | ExpectedInfluence | -0.26597 |
| 34 | OCI-R6 | Betweenness | 0.115321 | Closeness | 0.404776 | Strength | 0.617054 | ExpectedInfluence | -0.01867 |
| 35 | OCI-R7 | Betweenness | -1.04032 | Closeness | -1.20989 | Strength | -1.2077 | ExpectedInfluence | 0.002467 |
| 36 | OCI-R8 | Betweenness | -1.1083 | Closeness | -0.60538 | Strength | -0.56636 | ExpectedInfluence | -0.26488 |
| 37 | OCI-R9 | Betweenness | -0.97234 | Closeness | -1.62149 | Strength | -1.31844 | ExpectedInfluence | 0.056415 |
| 38 | OCI-R10 | Betweenness | -0.56447 | Closeness | 0.353268 | Strength | 0.261583 | ExpectedInfluence | -0.33115 |
| 39 | OCI-R11 | Betweenness | -0.42851 | Closeness | -0.29689 | Strength | 0.417925 | ExpectedInfluence | -0.36224 |
| 40 | OCI-R12 | Betweenness | -0.29255 | Closeness | -0.44559 | Strength | 0.018218 | ExpectedInfluence | 0.266845 |
| 41 | OCI-R13 | Betweenness | -0.22457 | Closeness | 0.635786 | Strength | 0.537371 | ExpectedInfluence | -0.67731 |
| 42 | OCI-R14 | Betweenness | 0.115321 | Closeness | 0.470306 | Strength | -0.0243 | ExpectedInfluence | 0.082547 |
| 43 | OCI-R15 | Betweenness | -0.29255 | Closeness | -0.32476 | Strength | -0.50031 | ExpectedInfluence | 0.266658 |
| 44 | OCI-R16 | Betweenness | -0.63245 | Closeness | -0.7953 | Strength | -0.80863 | ExpectedInfluence | -0.15586 |
| 45 | OCI-R17 | Betweenness | 0.727132 | Closeness | 0.902844 | Strength | 0.570499 | ExpectedInfluence | -0.31986 |
| 46 | OCI-R18 | Betweenness | -1.31224 | Closeness | -3.17574 | Strength | -2.93362 | ExpectedInfluence | -2.36963 |
| 47 | K1 | Betweenness | 0.115321 | Closeness | 0.753959 | Strength | 0.590768 | ExpectedInfluence | -0.83028 |
| 48 | K2 | Betweenness | -1.17628 | Closeness | -1.74359 | Strength | -2.00138 | ExpectedInfluence | -1.12138 |
| 49 | K3 | Betweenness | -0.49649 | Closeness | -0.63337 | Strength | -0.32787 | ExpectedInfluence | -0.07868 |
| 50 | K4 | Betweenness | -0.70043 | Closeness | -0.04059 | Strength | 0.071588 | ExpectedInfluence | -0.64434 |
| 51 | K5 | Betweenness | 0.047342 | Closeness | -0.24992 | Strength | 0.113729 | ExpectedInfluence | -1.57924 |
| 52 | K6 | Betweenness | -0.63245 | Closeness | -0.19178 | Strength | -0.18092 | ExpectedInfluence | -0.46185 |
| 53 | K7 | Betweenness | -1.24426 | Closeness | -1.47824 | Strength | -1.19291 | ExpectedInfluence | -0.4963 |
| 54 | K8 | Betweenness | -0.63245 | Closeness | -0.3257 | Strength | 0.207913 | ExpectedInfluence | 0.579277 |
| 55 | K9 | Betweenness | 0.047342 | Closeness | -0.25672 | Strength | -0.35857 | ExpectedInfluence | 0.300983 |
| 56 | K10 | Betweenness | -1.17628 | Closeness | -2.40658 | Strength | -1.7878 | ExpectedInfluence | -3.17154 |
